# The Macclesfield Infirmary Dispute

**Published:** 1902-01-04

**Authors:** 


### THE MACCLESFIELD INFIRMARY DISPUTE.

At a special meeting of the governors of the Macclesfield Infirmary held last Monday the Mayor read a letter from Miss Murdock Clarke, resigning her appointment as house surgeon. Miss Clarke, it is stated, resigned her position in order to relieve the governors of the difficult position in which they found themselves placed, and she left herself in their hands. The governors accepted the resignation and the Mayor proposed that she should have a solatium of a quarter's salary out of the infirmary funds. This suggestion was opposed, but a subscription was opened among the governors for a testimonial to Miss Clarke. A sum of £60 was realised, and it is intended, we believe, to increase the amount to £100. So we may presume this incident ends. Miss Clarke, it seems to us, thoroughly deserves the testimonial which is being raised in her behalf, and moreover she has our sympathy in that, from no fault of hers, beyond that of falling in with the fashion among the most advanced of the lady doctors, she has been made a scapegoat in a question in which she has no personal concern, For there seem to have been two points at issue at Macclesfield—one, whether the board or the staff were to nominate the house surgeon, the other, whether the house surgeon to a general hospital might properly be a woman. The first of these questions was not of the slightest concern to Miss Clarke, yet we can hardly doubt that this was the real point on which the medical staff felt called upon to join issue. For the board to have complicated the matter by appointing a lady was clearly a false move, which the staff took advantage of at once. They played their game with vigour, and they won, which is all right. It is the way with games. But so far as the question of employing women doctors as house surgeons in general hospitals is concerned we seem to be as we were. Miss Clarke retires, not because she is a woman, but to get the board out of a difficulty. The board accept her retirement, not because they confess that they were wrong in going against the opinion of the staff, but because the position had become intolerable. Nothing is decided either way, and so far as the woman question is concerned the whole matter is left in as great a fog as ever. The present position of this question is most unfair to those young ladies, of whom there is an increasing number every year, who being freshly qualified are anxious to realise the reward of all their labours. They are full of knowledge of their profession; they believe, and their successes at examinations encourage them in their belief, that they are quite equal to men in every particular; they find no rule laid down, no word of friendly warning given; and then when they accept appointments which place them in awkward positions people say hard things. It is unfair also to the governors of hospitals, who in opening such appointments to female candidates cannot tell whether they are offering a benefit or an insult. All of which is a great pity, and urgently demands that some general pronouncement on the subject should be made by those whose opinions would carry weight. It certainly is hard upon the younger medical women that it should be left for them individually to work out this question. Medical women, perhaps not without some reason, remembering the early history of the movement, are apt to regard male opinion regarding their sphere of work with a certain degree of suspicion; but that makes it all the more incumbent upon them that they should formulate some sort of collective opinion of their own in regard to such questions as these. If their leaders—for surely there are leaders among them to whose opinions the rest would look up with respect—decide that there are positions which medical women had better not try to occupy (we put it no stronger than that), we feel sure that young ladies who are just starting on their professional career would hesitate to act in opposition to such advice, while the governors of hospitals would have some guidance as to the sort of appointments they might with propriety throw open to female candidates and would act accordingly. If, on the other hand, the leaders among the medical women—ladies who from their position, their experience, and their knowledge of the world can speak with responsibility and some authority and whose opinion would certainly be received by medical men with respect—are prepared, after due consideration, to assert that in their opinion young medical women may with propriety do all the things that young medical men have to do, then, however much we may shrug our shoulders, the field will be cleared and, at the least, those junior lady practitioners who, acting in accordance with such advice, accept house-surgeoncies to general hospitals, will feel that in so doing they will have the support of their seniors in the somewhat difficult positions in which they may find themselves. These junior practitioners have, we think, a right to expect that they should be given either the support or the warning.

